# *Plasmodium berghei* LAPs form an extended protein complex that facilitates crystalloid targeting and biogenesis

**DOI:** 10.1016/j.jprot.2020.103925

**Published:** 2020-09-15

**Authors:** Annie Z. Tremp, Sadia Saeed, Vikram Sharma, Edwin Lasonder, Johannes T. Dessens

**Affiliations:** a Department of Infection Biology, Faculty of Infectious and Tropical Diseases, London School of Hygiene & Tropical Medicine, Keppel Street, London, WC1E 7HT, UK; b School of Biomedical Sciences, Faculty of Health, Plymouth University, Drake Circus, Plymouth, PL4 8AA, UK; c Department of Applied Sciences, Faculty of Life and Health Sciences, Northumbria University, Newcastle-Upon-Tyne, NE1 8ST, UK

## Abstract

Passage of malaria parasites through mosquitoes involves multiple developmental transitions, from gametocytes that are ingested with the blood meal, through to sporozoites that are transmitted by insect bite to the host. During the transformation from gametocyte to oocyst, the parasite forms a unique transient organelle named the crystalloid, which is involved in sporozoite formation. In *Plasmodium berghei*, a complex of six LCCL domain-containing proteins (LAPs) reside in the crystalloid and are required for its biogenesis. However, little else is known about the molecular mechanisms that underlie the crystalloid's role in sporogony. In this study, we have used transgenic parasites stably expressing LAP3 fused to GFP, combined with GFP affinity pulldown and high accuracy mass spectrometry, to identify an extended LAP interactome of some fifty proteins. We show that many of these are targeted to the crystalloid, including members of two protein families with CPW-WPC and pleckstrin homology-like domains, respectively. Our findings indicate that the LAPs are part of an intricate protein complex, the formation of which facilitates both crystalloid targeting and biogenesis.

**Significance:**

Reducing malaria parasite transmission by mosquitoes is a key component of malaria eradication and control strategies. This study sheds important new light on the molecular composition of the crystalloid, an enigmatic parasite organelle that is essential for sporozoite formation and transmission from the insect to the vertebrate host. Our findings provide new mechanistic insight into how proteins are delivered to the crystalloid, and indicate that the molecular mechanisms that underlie crystalloid function are complex, involving several protein families unique to *Plasmodium* and closely related organisms. The new crystalloid proteins identified will form a useful starting point for studies aimed at unravelling how the crystalloid organelle facilitates sporogony and transmission.

## Highlights

• A *Plasmodium berghei* LAP3 interactome of some 50 proteins was determined.
• Many proteins in the interactome constitute known or novel crystalloid proteins.
• The interactome includes protein families with PH-like and CPW-WPC domains.

## Graphical abstract

**Figure:**
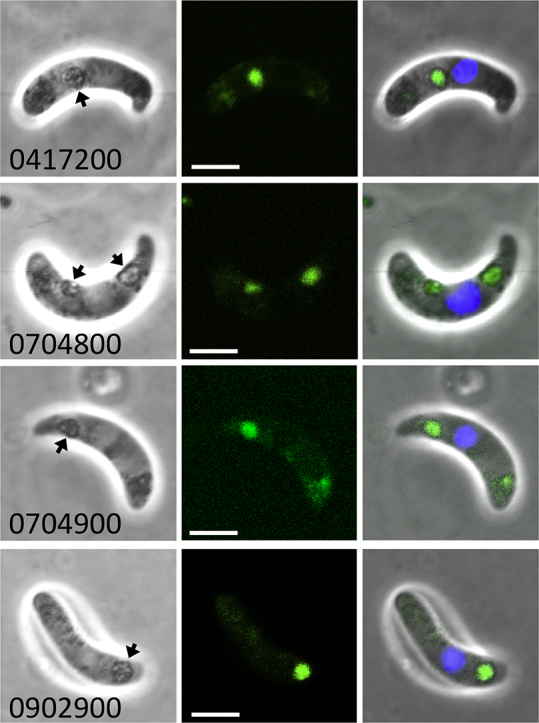

## 1 Introduction

Despite a significant fall in incidence rates in the last decade, malaria remains the most serious parasitic infection in humans. In 2018, the disease caused an estimated 228 million cases and 405,000 deaths, mostly in the under-fives. Malaria prophylaxis, treatment and control efforts continue to suffer from widespread resistance to anti-malarial drugs and insecticides, underpinning the urgent need for new therapies not only for prevention and treatment of the human disease, but also for reducing transmission, which will contribute to the global effort of malaria control and eradication. Malaria parasite transmission is initiated when *Anopheles* female mosquitoes ingest blood-borne gametocytes during blood feeding on a parasite-infected host. This sets off a rapid process of gamete formation and fertilization in the insect midgut. Zygotes undergo meiosis and transform into motile elongated forms called ookinetes that cross the midgut epithelium and round up to form young oocysts. In the following weeks, oocysts grow and divide by a process called sporogony, generating thousands of haploid sporozoites. These make their way to the insect's salivary glands to be transmitted to new hosts by mosquito bite. Parasites first multiply in liver cells to produce thousands of merozoites, which initiate new blood stage parasite infections. A small percentage of intraerythrocytic parasites transform into gametocytes to complete the life cycle.

In *P. berghei*, successful sporogony and sporozoite transmission requires expression of a family of six modular proteins rich in putative carbohydrate binding domains, named LCCL lectin adhesive-like proteins (LAPs) [[1], [2], [3], [4], [5], [6]]. The LAP-encoding genes are expressed in female gametocytes and are maternally inherited, and their gene products operate as a protein complex [3,[7], [8], [9]]. Another shared feature of the LAPs is their subcellular localization in the crystalloid, an unusual multivesicular organelle found exclusively in the ookinete and young oocysts life stages of the parasite [4,[10], [11], [12], [13]]. The crystalloid organelle forms after fertilization, during zygote transformation into ookinete and then oocyst, by a process of active transport and assembly of endoplasmic reticulum (ER)-derived vesicles [2]. Disruption of *lap* genes in *P. berghei* gives rise to similar loss-of-function phenotypes characterised by a failure of the oocysts to generate sporozoites [[1], [2], [3], [4],^14^].

Disruption or mutation of the LAPs affects formation of the crystalloids [2,4,7,15], identifying a link between LAP expression and crystalloid biogenesis. However, the role of the LAPs in sporozoite formation in the oocyst (sporogony) is much less clear. The LAPs are not expressed during sporozoite budding [4,14], and it is possible that their loss-of-function phenotype in the oocyst is in fact caused by the absence of the crystalloid and the broader protein repertoire contained within it, rather than by the absence of individual LAP molecules. This concept is supported by recent reports that null mutants of other crystalloid-resident proteins structurally and functionally unrelated to the LAPs, such as the S-acyl transferase DHHC10 and a membrane bound NAD(P) transhydrogenase (NTH), phenocopy the LAP null mutants [16,17]. This led to the hypothesis that the process of the crystalloid vesicles budding off the ER, a key step in crystalloid organelle formation [2], is at least partly dependent on the physical presence of the proteins contained within them (*i.e.* the crystalloid ‘cargo’) [2,18]. To shed further light on the underlying molecular mechanisms by which the crystalloids facilitate sporogony, we have here determined an extended LAP interactome and show that it contains many new crystalloid proteins. The biological significance of these findings is discussed.

## 2 Materials and methods

### 2.1 Parasite maintenance, culture, and purification

*P. berghei* ANKA clone 2.34 parasites were maintained as cryopreserved stabilates or by mechanical blood passage and regular mosquito transmission. Ookinete cultures were set up overnight from gametocytemic blood as previously described [19]. After 20-24 h, ookinetes were purified by ice-cold 0.17 M ammonium chloride lysis and centrifugation at 800 ×*g* for 15 min, followed by three washes in PBS with centrifugation at 500 ×*g.*

### 2.2 Immuno-affinity capture and *in vivo* crosslinking

Immuno-affinity capture of GFP fusion proteins was carried out by GFP pull-down using the μMACS GFP tagged protein isolation kit (Miltenyi Biotec) according to manufacturer's instructions. Briefly, 5–10 million purified parasites were lysed in 1 ml of pre-cooled lysis buffer (150 mM NaCl, 1% Triton X-100, 50 mM Tris-HCl pH 8.0) and incubated on ice for 30 min. Cell debris was removed by centrifugation at 10,000 ×*g*. Fifty μl anti-GFP microbeads were added to the supernatant and the mixture incubated on ice for 30 min to allow antibody binding. The cell lysate was then run by gravity through a magnetic microcolumn to capture the magnetic microbeads, followed by four 200 μl washes with lysis buffer. Proteins were eluted in 50 μl pre-heated 95 °C elution buffer (50 mM Tris-HCl pH 6.8, 50 mM DTT, 1% SDS, 1 mM EDTA, 0.005% bromophenol blue, 10% glycerol) and frozen until further use. For *in vivo* crosslinking, purified ookinetes were collected by low speed centrifugation (0.8 x*g*), resuspended in 0.5 ml PBS supplemented with 1% (*w*/*v*) paraformaldehyde and incubated at room temperature. The cells were collected by centrifugation after a total of 10 min in the fixative (including centrifugation), resuspended in 0.5 ml 250 mM Tris-HCl (pH 7.2), and incubated 10 min at room temperature to quench the formaldehyde. Cells were again collected by centrifugation followed by cell lysis and GFP pull-down as described.

### 2.3 Sample preparation for mass spectrometry

Four replicates each of crosslinked and non-crosslinked LAP3/GFP pulldown samples were analysed, as well as two replicates of LAP3-KO pulldown samples as negative controls. Protein samples in SDS sample buffer were digested with trypsin by a modified version of the filter-aided sample preparation (FASP) procedure of in solution digestion [20]. Samples were reduced in sample buffer with 100 mM DTT for 3 min at 95 before centrifuging them in Amicon Ultra filter tubes (30 kDa cut off) for 15 min at 16000 g and subsequent dilution of samples with 300ul of 50 mM ABC buffer. Samples were centrifuged once more to ensure maximal removal of DTT and SDS followed by alkylation step with 50 mM of 2-chloroacetamide (Sigma). Single step overnight trypsin digestion at enzyme to substrate ratio of 1:100 was carried out at 37 °C. Tryptic digests were acidified to a final concentration of 0.1% TFA and purified by STAGE tips [21].

### 2.4 Liquid chromatography tandem mass spectrometry

Peptide digest samples were analysed by an LC-MS/MS platform composed of the Ultimate 3000 UPLC (Thermo Fisher, Germany) connected to the Orbitrap Velos Pro mass spectrometer (Thermo Fisher, Germany) for acquiring tandem mass spectrometry data. Peptide samples were loaded on a 2 cm Acclaim™ PepMap™100 Nano-Trap Column (Thermo Fisher, Germany) and were separated by a 25 cm Acclaim™ PepMap™100 Nano LC column (Thermo Fisher, Germany) packed with 3 μm C18 beads with a flow-rate of 300 nl/min in a 120 min gradient of 95% buffer A/5% buffer B to 65% buffer A /35% buffer B (buffer A: 0.5% acetic acid. Buffer B: 0.5% acetic acid in 100% acetonitrile). Peptides eluting from the column were ionised and injected into the mass spectrometer at 2.3 kV spray voltage. The Orbitrap mass spectrometer operated in a data-dependent mode and switched between MS and MS2 automatically by a top 10 method. The Orbitrap cell acquired full-scan spectra of intact peptides (*m*/*z* 350–1500) with automated gain control accumulation value of 1.000.000 ion and with a resolution of 60.000. The ten most abundant ions were sequentially isolated and fragmented in the linear ion trap, where dissociation was induced through collision, using an accumulation target value of 10.000, a normalized collision energy of 35% and a capillary temperature of 275 °C. Dynamic exclusion of ions sequenced within the 45 previous seconds was applied. Unassigned charge states and singly charged ions were excluded from sequencing. For MS2 selection, a minimum of 10.000 counts was required.

### 2.5 Protein identification and quantification

Tandem mass spectrometry data was searched by Andromeda [22] search engine integrated in MaxQuant (Version 1.5.3.8) [23] for protein identification. Peak lists were generated for the top 12 most intense MS peaks in 100 Da windows by MaxQuant prior to the database search. The protein database contained protein sequences from *P. berghei* (http://plasmodb.org/common/downloads/release-29/PbergheiANKA/fasta/data/) and from mouse (http://www.uniprot.org/downloads, downloaded at 2 November 2016) supplemented with frequently observed contaminants. Andromeda search parameters for protein identification were set to tolerance of 6 ppm for the parental peptide and 0.5 Da for fragmentation spectra and trypsin specificity allowing up to 2 miscleaved sites. Deamination of glutamine, oxidation of methionine, and asparagine and protein N-terminal acetylation were set as variable modifications, carboxyamidomethylation of cysteines was specified as a fixed modification. Minimal required peptide length was specified at 7 amino acids. Peptides and proteins detected with a false discovery rate (FDR) of 1% were accepted. Excluded from validation were proteins identified by site only, external contaminants and reversed proteins. Proteins were quantified by normalized summed peptide intensities [24] computed in MaxQuant with the label free quantification (LFQ) option switched on. Hierarchical clustering of relative LFQ profiles ranging from 0 to 1 was performed in Perseus 1.3.7.1 [25].

### 2.6 Bioinformatics filters

Protein outputs were subjected to the following bioinformatics filters: (1) Absence of a predicted amino-terminal ER signal peptide (SP-HMM algorithm); (2) Presence of a predicted carboxy-terminal ER retention signal (XDEL) for predicted luminal proteins (one letter amino acid code, X = any amino acid); (3) Transcript level ratio gametocytes/asexual stages ≤1 [26]; (4) Transcript level ratio female gametocyte/male gametocyte ≤1 [27].

### 2.7 Data availability

The mass spectrometry proteomics data have been deposited to the ProteomeXchange Consortium (http://proteomecentral.proteomexchange.org) *via* the PRIDE partner repository [28] with the dataset identifier PXD019454.

### 2.8 Animal experiments

All laboratory animal work was carried out in accordance with the Arrive guidelines and the United Kingdom Animals (Scientific Procedures) Act 1986 implementing European Directive 2010/63/EU for animal experiments. Experiments were generally conducted in 6–8 weeks old female CD1 mice, specific pathogen free and maintained in filter cages. Animal welfare was assessed daily and animals were humanely killed upon reaching experimental or clinical endpoints. Mice were infected with parasites suspended in phosphate buffered saline (PBS) by intraperitoneal injection, or by infected mosquito bite on anaesthetized animals. Intra-erythrocytic parasitemia was monitored regularly by collecting of a small volume of blood from a superficial tail vein. Drugs were administered by intraperitoneal injection or where possible were supplied in drinking water. Parasitized blood was harvested by cardiac bleed under general anaesthesia without recovery.

### 2.9 Generation of CPW-WPC protein targeting constructs

An approximately 2.2 kb fragment corresponding to the coding sequence and 5’UTR of PBANKA_0943400 was PCR amplified from *P. berghei* genomic DNA with primers CPW1-F (TTGGGCTGCAGTCGAGCAAGGGACTGTAATGGTGA) and CPW1-R (ATGAGGGCCCCTAAGCTCTCTAAGGTGATCCCTTTTTTGTTTG) and cloned into *Sal*I/*Hin*dIII-digested plasmid pBS-EGFP-hDHFR to give pBS-CPW1/GFP. The plasmid was linearized with *Hin*dIII before gene targeting by single crossover homologous recombination.

An approximately 1.4 kb fragment corresponding to the coding sequence and 5’UTR of PBANKA_1449300 was PCR amplified from *P. berghei* genomic DNA with primers CPW2-F (TTGGGCTGCAGTCGAGATAACAATTGAACTTGGTAAAGTAGCA) and CPW2-R (ATGAGGGCCCCTAAGCTCAATTTTTGAACTTGTATAAAAGAATAATTAATTT) and cloned into *Sal*I/*Hin*dIII-digested plasmid pBS-EGFP-hDHFR to give pBS-CPW2/GFP. The plasmid was linearized with *Hin*dIII before gene targeting by single crossover homologous recombination.

An approximately 2.4 kb fragment corresponding to the coding sequence and 5’UTR of PBANKA_1218300 was PCR amplified from *P. berghei* genomic DNA with primers CPW3-F (TTGGGCTGCAGTCGAGCAATATGGGATTGCGATTTG) and CPW3-R (ATGAGGGCCCCTAAGCTCACATCGATTATTGCCCCTG) and cloned into *Sal*I/HindIII-digested plasmid pBS-EGFP-hDHFR to give pBS-CPW3/GFP. The plasmid was linearized with *Blp*I before gene targeting by single crossover homologous recombination.

An approximately 1.5 kb fragment corresponding to the coding sequence and 5’UTR of PBANKA_1015400 was PCR amplified from *P. berghei* genomic DNA with primers CPW4-F2 (TTGGGCTGCAGTCGAGACAATTTTTTATTGTTAAAATAGATAATGG) and CPW4-R (ATGAGGGCCCCTAAGCTGATAACAAGGTTTGAAACTATTTCCCC) and cloned into *Sal*I/*Hin*dIII-digested plasmid pBS-EGFP-hDHFR to give pBS-CPW4/GFP. The plasmid was linearized with *Cla*I before gene targeting by single crossover homologous recombination.

### 2.10 Generation of PHL protein targeting constructs

An approximately 1.5 kb fragment corresponding to the coding sequence and 5’UTR of PBANKA_0417200 was PCR amplified from *P. berghei* genomic DNA with primers PH1-F (TTGGGCTGCAGTCGAGGTACCACAAAACAATTGTCATAAAATAGTTCTTG) and PH1-R (ATGAGGGCCCCTAAGCTCATATCGTTATCGTTTTCTTCATTG) and cloned into *Sal*I/*Hin*dIII-digested plasmid pBS-EGFP-hDHFR to give pBS-PH1/GFP. The plasmid was linearized with *Hin*dIII before gene targeting by single crossover homologous recombination.

An approximately 1.8 kb fragment corresponding to the coding sequence and 5’UTR of PBANKA_0704800 was PCR amplified from *P. berghei* genomic DNA with primers PH2-F (TTGGGCTGCAGTCGAGGTACCATGCGCATTTATAATATACATAAATAAG) and PH2-R (ATGAGGGCCCCTAAGCTCAAATTATCATCATCATTATCTTCATATTCTTC) and cloned into *Sal*I/*Hin*dIII-digested plasmid pBS-EGFP-hDHFR to give pBS-PH2/GFP. The plasmid was linearized with *Cla*I before gene targeting by single crossover homologous recombination.

An approximately 2.0 kb fragment corresponding to the coding sequence and 5’UTR of PBANKA_0704900 was PCR amplified from *P. berghei* genomic DNA with primers PH3-F (TTGGGCTGCAGTCGAGGTACCATTTCTTATTAATAGACAAAACAAAAATAAT) and PH3-R (ATGAGGGCCCCTAAGCTCTTAAGAGAAATATTTGGATTACTGCTTTT) and cloned into *Sal*I/*Hin*dIII-digested plasmid pBS-EGFP-hDHFR to give pBS-PH3/GFP. The plasmid was linearized with *Nhe*I before gene targeting by single crossover homologous recombination.

An approximately 1.8 kb fragment corresponding to the coding sequence and 5’UTR of PBANKA_0902900 was PCR amplified from *P. berghei* genomic DNA with primers PH4-F (TTGGGCTGCAGTCGAGGTACCTTTGTACATACATTCAAAAGGCG) and PH4-R (ATGAGGGCCCCTAAGCTGGTCTCTGCTTTTATGGAAACTAAAAAA) and cloned into *Sal*I/*Hin*dIII-digested plasmid pBS-EGFP-hDHFR to give pBS-PH4/GFP. The plasmid was linearized with *Cla*I before gene targeting by single crossover homologous recombination.

### 2.11 Generation of TPM2 targeting construct

The entire coding sequence of PBANKA_1104100 plus *ca.* 0.6 kb of upstream sequence was PCR amplified from genomic DNA with primers pDNR-110410-F (ACGAAGTTATCAGTCGAGGTACCGCTCAAACTATTCCTCCTCAATC) and pDNR-110410-R (ATGAGGGCCCCTAAGCTGTTTATTCTATATACAACAGTGATTAAATATACAATG) and cloned into *Sal*I/*Hin*dIII-digested pDNR-EGFP by in-fusion cloning to give plasmid pDNR-TPM2/GFP. The 3'UTR of of this gene was amplified with primers pLP-110410-F (ATATGCTAGAGCGGCCATGTATGATATGTATTTTTTGCG) and pLP-110410-R (CACCGCGGTGGCGGCCAATTAAATGAAACTGCGGCAC) and the resulting fragment cloned into *Not*I-digested pLP-hDHFR by in-fusion cloning to give plasmid pLP-hDHFR/TPM2. The *tpm2*-specific sequence from pDNR-TPM2/GFP was transferred to pLP-hDHFR/TPM2 by Cre/loxP recombination to give the final construct pLP-TPM2/GFP. This plasmid was digested with *Kpn*I and *Sac*II before gene targeting by double crossover homologous recombination.

### 2.12 Generation of parasite lines

Parasite transfection, pyrimethamine selection and dilution cloning were performed as previously described [29,30]. Genomic DNA extraction for diagnostic PCR was performed as previously described [31].

### 2.13 PCR

PCR amplification was carried out with custom oligonucleotide primers using Advantage 2 polymerase or Advantage HD polymerase (Takara Bio) on a thermal cycler typically with 30s denaturation at 94 °C, 30s annealing at 50 °C, and 1 min per kb elongation at 62 °C.

### 2.14 Microscopy

Live parasite samples were assessed, and images captured, using a Zeiss LSM510 or LSM880 laser scanning confocal microscope using 100× oil objectives and Zeiss Image Browser or ZEN 3.0 software.

## 3 Results

### 3.1 *Plasmodium* LAPs are part of an extended interactome

We previously studied molecular interactions between the six LAPs using a series of genetically modified parasite lines stably expressing LAPs individually fused to green fluorescent protein (GFP), combined with GFP affinity purification and label free quantitative mass spectrometry (AP-MS) [7]. This revealed that LAP1, LAP2 and LAP3 form strong interactions with each other and readily co-purify. In contrast, LAP4, LAP5 and LAP6 associated more weakly and were only pulled down with the rest of the LAP complex after *in vivo* crosslinking [7]. To identify other proteins interacting with the LAP complex and discover potential new crystalloid proteins, we made assumptions that such proteins would behave like LAPs 4–6 in GFP pulldown experiments, and would also have similar features to LAPs 4–6 with regards to their expression and subcellular trafficking in the parasite.

**Fig. 1:**
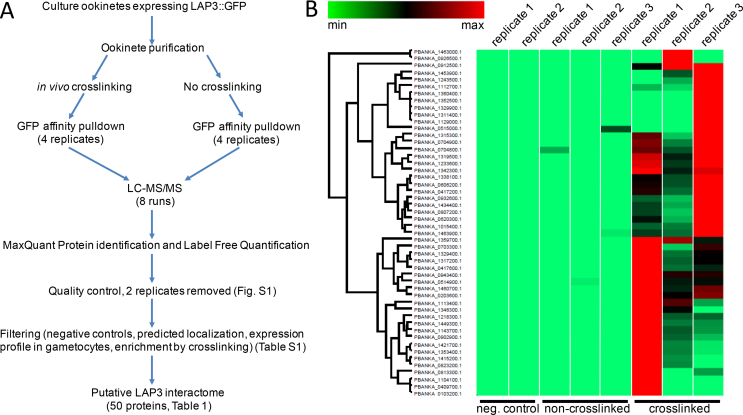
Proteomics approach used to determine the LAP3 interactome. A: Workflow of the different experimental steps used in the analysis (see Materials and Methods for details). B: Hierarchical clustering of relative LFQ profiles of the putative LAP3 interactome identified (see Table 1 for annotation). Replicate GFP pulldown samples from *Plasmodium berghei* ookinetes expressing GFP-tagged LAP3 with or without *in vitro* crosslinking before pulldown are included, as well as two negative controls obtained from LAP3-KO parasites. LAP3 interactome proteins are largely absent in the non-crosslinked samples and negative controls.

**Table 1:** Putative LAP3 interactome of *Plasmodium berghei.*

	Protein ID	Annotation	MG-SG transition^1^	Significantly reduced	Reference
1	PBANKA_1319500	LCCL domain-containing protein (LAP4)	−0.92	No power	[12]
2	PBANKA_1315300	LCCL domain-containing protein (LAP5)	−1.57	No power	[12]
3	PBANKA_0417200	PH domain-containing protein	n/a	n/a	[43]
4	PBANKA_0704900	Crystalloid-specific PH domain-containing protein, putative	−0.29	No	[32]
5	PBANKA_0704800	Conserved Plasmodium protein, unknown function	n/a	n/a	
6	PBANKA_1233600	Secreted ookinete protein, putative (PSOP13)	−2.14	Yes	[35]
7	PBANKA_0912500	Conserved Plasmodium protein, unknown function	−0.23	No	
8	PBANKA_0943400	CPW-WPC family protein	−0.77	No power	[33]
9	PBANKA_0606200	Blood stage antigen 41–3 precursor, putative	−1.07	No power	
10	PBANKA_0203600	Conserved Plasmodium protein, unknown function	n/a	n/a	
11	PBANKA_0417600	LCCL domain-containing protein (LAP6)	n/a	n/a	[12]
12	PBANKA_1342300	Conserved Plasmodium protein, unknown function	0.01	No	
13	PBANKA_1317200	Pyridine nucleotide transhydrogenase, putative (NTH)	−2.41	Yes	
14	PBANKA_0807200	Conserved Plasmodium protein, unknown function (POM7)	0.19	No	[45]
15	PBANKA_0620300	Conserved Plasmodium protein, unknown function	−1.77	Yes	
16	PBANKA_1453900	Conserved Plasmodium protein, unknown function	n/a	n/a	
17	PBANKA_1449300	CPW-WPC family protein	n/a	n/a	[33]
18	PBANKA_0703300	Conserved Plasmodium protein, unknown function	−0.37	No	
19	PBANKA_0514900	28 kDa ookinete surface protein (P28)	−0.27	No	[39]
20	PBANKA_1460700	Dipeptidyl aminopeptidase 2 (DPAP2)	−0.26	No	
21	PBANKA_0926500	Petidase, M16 family, putative	n/a	n/a	
22	PBANKA_1421700	Secreted ookinete protein, putative (PSOP20)	n/a	n/a	[35]
23	PBANKA_1015400	CPW-WPC family protein	n/a	n/a	[33]
24	PBANKA_1463900	HSP20-like chaperone, putative	n/a	n/a	
25	PBANKA_1329400	Conserved Plasmodium protein, unknown function	−0.44	No power	
26	PBANKA_1112700	Conserved Plasmodium protein, unknown function	−0.75	No power	
27	PBANKA_1218300	CPW-WPC family protein	−0.79	No power	[33]
28	PBANKA_0932600	Conserved Plasmodium protein, unknown function	−1.14	Yes	
29	PBANKA_1353400	Secreted ookinete protein, putative (PSOP7)	n/a	n/a	[35]
30	PBANKA_1434400	Secreted ookinete protein, putative (PSOP17)	−1.25	No power	[35]
31	PBANKA_1415200	Conserved Plasmodium protein, unknown function	n/a	n/a	
32	PBANKA_1143700	Secreted ookinete protein, putative (PSOP2)	−1.57	Yes	[35]
33	PBANKA_1243500	Conserved Plasmodium protein, unknown function	n/a	n/a	
34	PBANKA_1338100	Conserved Plasmodium protein, unknown function	−1.74	Yes	
35	PBANKA_1329900	Conserved Plasmodium protein, unknown function	n/a	n/a	
36	PBANKA_1360400	Conserved Plasmodium protein, unknown function	0.17	No	
37	PBANKA_0902900	Conserved Plasmodium protein, unknown function	n/a	n/a	
38	PBANKA_1346300	CPW-WPC family protein	−0.55	No	[33]
39	PBANKA_0103200	Conserved Plasmodium protein, unknown function	n/a	n/a	
40	PBANKA_0409700	Plasmepsin VI	n/a	n/a	[35]
41	PBANKA_1129000	Secreted ookinete protein, putative (PSOP6)	−1.55	Yes	[35]
42	PBANKA_1359700	6-cysteine protein (P47)	−0.07	No	[38]
43	PBANKA_0823200	Conserved Plasmodium protein, unknown function	−0.30	No	
44	PBANKA_1463000	Osmiophilic body protein G377	−0.71	No power	[37]
45	PBANKA_1104100	MOLO1 domain-containing protein, putative (TPM2)	−2.17	Yes	
46	PBANKA_1352500	CPW-WPC family protein	−1.27	No power	[33]
47	PBANKA_1113400	Secreted ookinete protein, putative (PSOP12)	0.11	No	[35]
48	PBANKA_0813300	Conserved Plasmodium protein, unknown function	n/a	n/a	
49	PBANKA_0515000	25 kDa ookinete surface antigen precursor (P25)	−0.42	No	[39]
50	PBANKA_1311400	Conserved Plasmodium protein, unknown function	−0.26	No	

1 Log2-fold change in transition from midgut oocyst to salivary gland sporozoite in pools of null mutant parasites as assessed by [36].

**Table 2:** Putative TPM2 interactome of *Plasmodium berghei.*

	Protein ID	Annotation
1	PBANKA_1143700	Secreted ookinete protein, putative (PSOP2)
2	PBANKA_1353400	Secreted ookinete protein, putative (PSOP7)
3	PBANKA_1300700	LCCL domain-containing protein (LAP2)
4	PBANKA_1319500	LCCL domain-containing protein (LAP4)
5	PBANKA_1035200	LCCL domain-containing protein (LAP1)
6	PBANKA_0204500	LCCL domain-containing protein (LAP3)
7	PBANKA_0704800	Conserved Plasmodium protein, unknown function
8	PBANKA_0515000	25 kDa ookinete surface antigen precursor (P25)
9	PBANKA_0417200	PH domain-containing protein
10	PBANKA_0514900	28 kDa ookinete surface protein (P28)
11	PBANKA_1317200	Pyridine nucleotide transhydrogenase, putative (NTH)
12	PBANKA_0203600	Conserved Plasmodium protein, unknown function
13	PBANKA_1233600	Secreted ookinete protein, putative (PSOP13)
14	PBANKA_0704900	Crystalloid-specific PH domain-containing protein, putative
15	PBANKA_0807200	Conserved Plasmodium protein, unknown function (POM7)
16	PBANKA_0902900	Conserved Plasmodium protein, unknown function
17	PBANKA_0417600	LCCL domain-containing protein (LAP6)
18	PBANKA_1359700	6-cysteine protein (P47)
19	PBANKA_0620300	Conserved Plasmodium protein, unknown function
20	PBANKA_1329400	Conserved Plasmodium protein, unknown function
21	PBANKA_0926500	Peptidase, M16 family, putative
22	PBANKA_0412900	Circumsporozoite- and TRAP-related protein (CTRP)
23	PBANKA_1315300	LCCL domain-containing protein (LAP5)
24	PBANKA_1218300	CPW-WPC family protein
25	PBANKA_1463900	Conserved Plasmodium protein, unknown function
26	PBANKA_1352500	CPW-WPC family protein
27	PBANKA_1342300	Conserved plasmodium protein, unknown function
28	PBANKA_0943400	CPW-WPC family protein, putative
29	PBANKA_0825900	Conserved Plasmodium protein, unknown function
30	PBANKA_0912500	Conserved Plasmodium protein, unknown function
31	PBANKA_1432300	Cell traversal protein for ookinetes and sporozoites (CelTOS)

For this study, AP-MS was carried out with ookinetes expressing GFP-tagged LAP3 (LAP3::GFP) that were prepared with and without *in vivo* crosslinking (Fig. 1A). This was initially carried out with four biological replicates each, but following a quality control step assessing proteome coverage and replicate reproducibility one replicate in each group was removed because of poor sequence coverage, improving correlation between replicates (Fig. S1). Protein identification and label free quantitation of the remaining replicates resulted in an initial data set of 273 proteins that was subjected to filtering procedures for eliminating non-specific interactions (Fig. 1A). As a first filter, proteins were removed that were present in negative control samples from GFP-expressing LAP3 knockout parasites (LAP3-KO [2]) (Table S1). Because the LAPs are trafficked to the crystalloid *via* the ER and possess an amino-terminal ER signal peptide sequence [2,10], we also eliminated proteins that either lacked a predicted ER signal peptide, or that possessed a carboxy-terminal ER retention signal (Table S1). In addition, because the LAP-encoding genes are expressed in gametocytes and show no discernible expression in asexual blood stages [12], proteins whose transcription was not upregulated in sexual compared to asexual blood stages were also eliminated (Table S1). Because the LAP-encoding genes are predominantly transcribed in female gametocytes, proteins whose transcript levels were not upregulated in female compared to male gametocytes were also removed (Table S1). Finally, we removed proteins that were less than 2-fold enriched in the crosslinked samples (Table S1). These combined filters resulted in a putative LAP3 interactome of 50 proteins (Table 1, Fig. 1B). As expected, the reference proteins LAPs 4‐6 were present in this interactome (Table 1), because they pull down with LAP3 only after *in vivo* crosslinking [7]. Also as expected, LAPs 1‐3 were not identified by this analysis (Table 1), because these proteins are also pulled down from non-crosslinked samples [7].

We further scrutinized the suitability of our experimental approach in identifying a genuine interaction network by performing a reciprocal AP-MS experiment with the newly identified putative interactor PBANKA_1104100 (Table 1), here named TPM2. To do so, ookinetes expressing GFP-tagged TPM2 were cultured and purified and then subjected to AP-MS with and without prior crosslinking. This identified an initial set of 235 proteins (Table S2), which after applying the same set of bioinformatics filters yielded a TPM2 interactome of 31 proteins (Table 2). Even though this interactome was smaller than that identified for LAP3, probably because of the lower number of replicates analysed, 27 of its 31 proteins (87%) overlapped with the LAP3 interactome. As expected, all six LAPs were identified (Table 2). This is because the bait protein TPM2 does not form strong interactions with the LAPs, and hence these proteins are only pulled down after *in vivo* crosslinking. The strong overlap between the TPM2 and LAP3 interactomes provides supporting evidence that they reflect a genuine protein interaction network.

### 3.2 The LAP interactome is enriched in crystalloid proteins

**Fig. 2:**
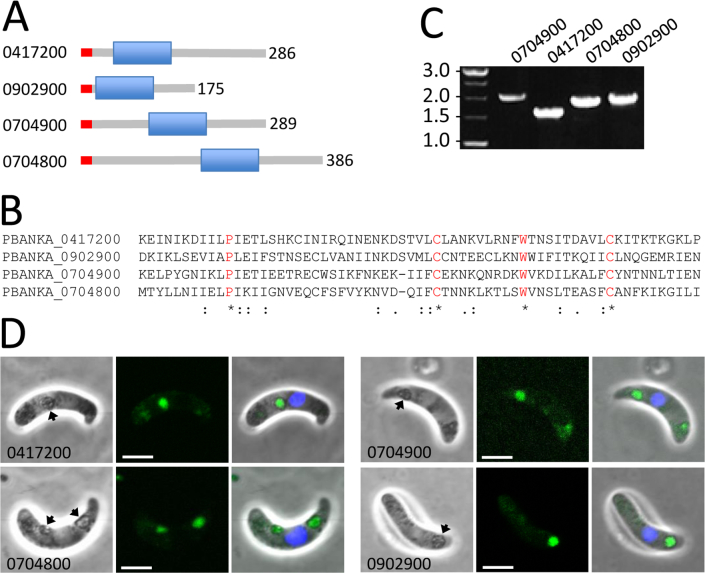
Characterization of pleckstrin homology-like (PHL) domain proteins PBANKA_0417200, PBANKA_0704800, PBANKA_0704900 and PBANKA_0902900. A: Schematic diagram of protein structures with relative positions of the PHL domains and ER signal peptides (red). Protein lengths (amino acids) are indicated on the right-hand side. B: Alignment of the shared domain reveals a unique amino acid signature C(X)_9_W(X)_9_C (one letter amino acid code, X = any amino acid). C: PCR diagnostic for integration of the GFP-tagged alleles into the target loci gives rise to the expected products of approximately 1.6 kb (PBANKA_0417200, primers PH1–5’diag (TTATATAATAAATCCTAACACTTCATCG) and GFP-R (GTGCCCATTAACATCACC)); 1.9 kb (PBANKA_0704800, primers PH2–5’diag (AAAGTATGAACGCATTAAAAAAATC) and GFP-R); 2.1 kb (PBANKA_0704900, primers PH3–5’diag (TACAGGTAAAAAAGATTGGCAT) and GFP-R); and 2.0 kb (PBANKA_0902900, primers PH3–5’diag (CGATTTTACATTTACTATTTTGTTAAAAAG) and GFP-R). D: Brightfield and fluorescence confocal images of ookinetes expressing GFP-tagged PBANKA_0417200, PBANKA_0704800, PBANKA_0704900 and PBANKA_0902900, showing localisation in focal spots associated with pigment (arrows) characteristic of crystalloids. Hoechst DNA staining (blue) labels nuclei. (For interpretation of the references to colour in this figure legend, the reader is referred to the web version of this article.)

By analogy to LAP4, LAP5 and LAP6 (Table 1), a significant proportion of proteins of the extended LAP3 interactome were expected to localise in the crystalloid organelle. Indeed, our analysis identified PBANKA_1317200, a recently characterised membrane-bound NAD(P) transhydrogenase (NTH) that resides in the crystalloid organelle in *P. berghei* [17]. The analysis also identified PBANKA_0704900, a putative pleckstrin homology (PH) domain-containing protein whose orthologue in *P. yoelii* was recently shown to reside in the crystalloids [32]. Using this protein's amino acid sequence in BLAST homology searches, we identified three paralogues in the *Plasmodium* genome, namely PBANKA_0417200, 0902900 and 0704800 (Fig. 2A). These were also present in the LAP3 interactome (Table 1). The genes for two of these proteins are in fact located tandemly on chromosome 7, pointing to a relatively recent gene duplication event. Their shared domain only has weak homology with the archetypal PH domain, and in fact contains a unique amino acid signature C(X)_9_W(X)_9_C (one letter amino acid code, X = any amino acid) (Fig. 2B). We therefore propose the name PH-like (PHL) domain-containing proteins for this group of molecules. GFP tagging of PBANKA_0704900 in transgenic parasites confirmed its localisation in the crystalloid of *P. berghei* ookinetes (Fig. 2CD), consistent with the close relationship between these rodent malaria parasite species. Upon GFP tagging in transgenic lines, the other three family members also displayed a localisation pattern in ookinetes consistent with crystalloids (Fig. 2CD). Thus, the PHL domain-containing proteins appear to constitute a novel crystalloid-specific protein family.

**Fig. 3:**
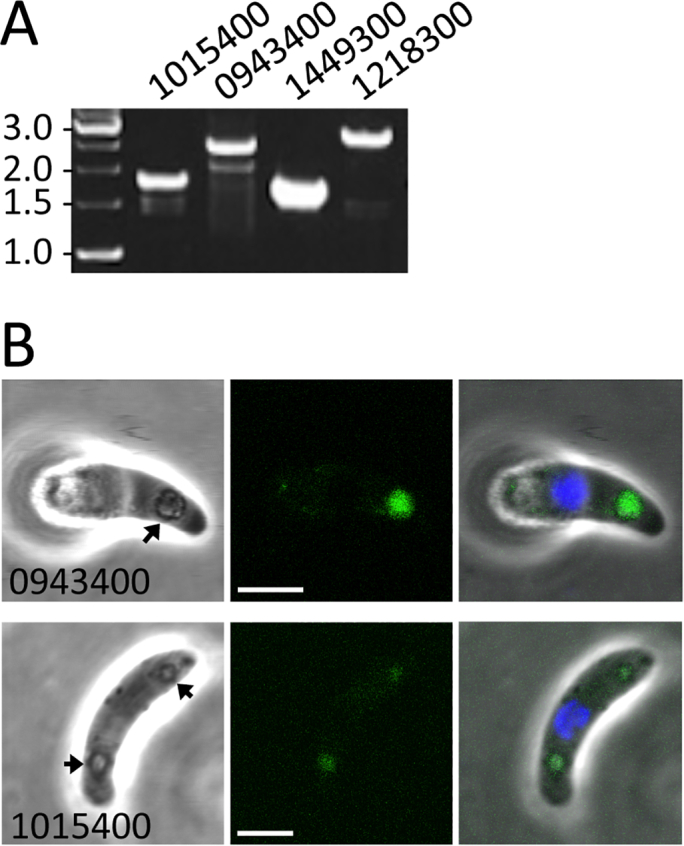
Characterization of CPW-WPC domain proteins PBANKA_1015400, PBANKA_0943400, PBANKA_1449300 and PBANKA_1218300. A: PCR diagnostic for integration of the GFP-tagged allelles into the target loci gives rise to the expected products of approximately 1.7 kb (PBANKA_1015400, primers CPW4–5’diag (AAGACAGTAAATACAATCCATAGGTC) and GFP-R (GTGCCCATTAACATCACC)); 2.4 kb (PBANKA_0943400, primers CPW1–5’diag (CCATATTATGACTTTCGAACCC) and GFP-R); 1.6 kb (PBANKA_1449300, primers CPW2–5’diag (CTTACACAAAATGGTATAAACAATTTTTC) and GFP-R); and 2.7 kb (PBANKA_1218300, primers CPW3–5’diag (CGAGTCCGAAAAGGTATACATATG) and GFP-R). B: Brightfield and fluorescence confocal images of ookinetes expressing GFP-tagged PBANKA_1015400 and PBANKA_0943400, showing localisation in focal spots associated with pigment (arrows) characteristic of crystalloids. GFP-tagged PBANKA_1449300 and PBANKA_1218300 did not show discernible GFP fluorescence (not shown). Hoechst DNA staining (blue) labels nuclei. (For interpretation of the references to colour in this figure legend, the reader is referred to the web version of this article.)

Our analysis identified a further two proteins, namely PBANKA_1352500 and 1346300 (Table 1), which were previously reported to be crystalloid-resident by GFP tagging [33]. These two proteins are part of the nine-member ‘CPW-WPC’ domain-containing protein family [33,34]. Interestingly, an additional four members of this family were identified by our analysis of the LAP3 interactome, namely PBANKA_0943400, 1015400, 1449300 and 1218300 (Table 1). We generated GFP-tagged parasite lines for the latter four CPW-WPC proteins (Fig. 3A), which revealed that PBANKA_0943400 and 1015400, too, displayed weak GFP signal with a crystalloid-like distribution in ookinetes (Fig. 3B). Although we could not detect discernible GFP signal in ookinetes of the other two parasite lines, possibly due to low expression levels (data not shown), our findings indicate that at least several CPW-WPC family members are involved with the crystalloid.

**Fig. 4:**
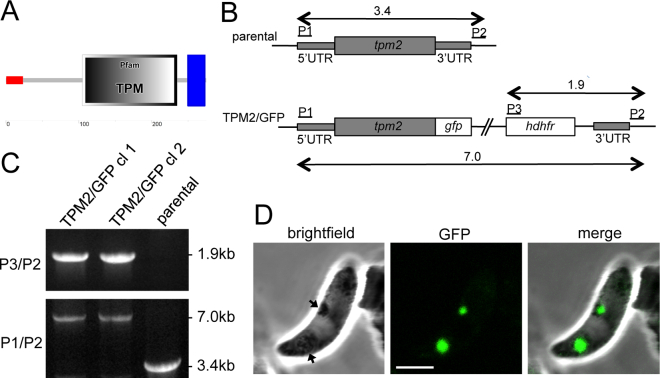
Characterization of TPM domain protein PBANKA_1104100 (TPM2). A: Predicted structure showing ER signal peptide (red), TPM domain and C-terminal transmembrane domain (blue), produced with Simple Modular Architecture Research Tool (http://smart.embl-heidelberg.de). B: Schematic diagram of the unmodified (parental) and modified *tpm2* allele in parasite lines TPM2/GFP. The *tpm2* gene is indicated with coding sequence (wide grey bars, introns not shown) and 5′ and 3′ untranslated regions (UTRs) (narrow grey bars). Also indicated are the relative positions of the GFP module (*gfp*); the human DHFR selectable marker gene cassette (*hdhfr*); and primers used for diagnostic PCR amplification (P1-P3). Primer P2 sequence is not present within the targeting vector. Sizes of PCR products are also indicated. C: Diagnostic PCR for integration into the target locus with primers P3 (ACAAAGAATTCATGGTTGGTTCGCTAAACT) and P2 (CATCTTGAGGTATTTGTGCATATTC), giving rise to a 1.9 kb product (top panel). Diagnostic PCR with primer pair P1 (ACGAAGTTATCAGTCGAGGTACCGCTCAAACTATTCCTCCTCAATC) and P3 amplified an approximately 3.4 kb fragment from the parental WT parasites, and a 7.0 kb fragment in the TPM2/GFP parasites, confirming absence of the unmodified allele in the transgenic parasite line (bottom panel). D: Brightfield and GFP fluorescence images of a live ookinete of parasite line TPM2/GFP, showing localisation in focal spots associated with pigment (arrows) characteristic of crystalloids. (For interpretation of the references to colour in this figure legend, the reader is referred to the web version of this article.)

By analogy to LAP4, LAP5 and LAP6 (Table 1), a proportion of proteins of the extended LAP3 interactome was also expected to phenocopy the LAP null mutants. Indeed, NTH (PBANKA_1317200) was shown to be required for crystalloid biogenesis and sporozoite formation in *P. berghei* [17]. Equally, our analysis identified PBANKA_1233600 (PSOP13) and PBANKA_0409700 (plasmepsin VI) (Table 1), both of which have reported loss-of-function phenotypes characterised by a lack of sporozoite development in the oocyst [35], which is consistent with a potential crystalloid localisation. A recent genome-scale barcode study analysed the contribution of over 1300 *P. berghei* genes through the life cycle in pools of gene knockout parasites [36]. Whilst this type of analysis proved useful to identify genes essential for liver stage parasite development, it was shown to be much less suitable to identify genes that are essential during the diploid and polyploid life cycle stages (*i.e.* zygotes, ookinetes and oocysts) due to heterozygous rescue [36]. Nonetheless, in this barcode analysis NTH and PSOP13 null mutants (plasmepsin VI was not assessed) displayed approximately 4- to 5-fold reductions, respectively, in their transition from midgut oocyst to salivary gland sporozoite (Table 1) [36]. Whilst these levels of reduction fall well short of the actual reductions (close to 100%) in oocyst to salivary gland sporozoite transition that are observed when the null mutants are assessed on their own [17,35], they could reflect their null mutant phenotypes in a context of heterozygous rescue within the mixed population of null mutants. When we assessed the other proteins in the LAP3 interactome by this analysis, a further six proteins were identified that displayed statistically significant and greater than 2-fold reductions in oocyst to salivary gland sporozoite conversion upon knockout, namely PBANKA_0620300, 0932600, 1338100, 1143700, 1129000, and 1104100 (Table 1) [36]. These are prime candidates to constitute additional crystalloid proteins. To test this hypothesis, we generated a parasite line expressing a GFP-tagged version of PBANKA_1104100, named TPM2/GFP (Fig. 4). This protein possesses a central TPM domain named after its founding proteins TLP18.3, Psb32 and MOLO-1 (Pfam 04536), as well as a transmembrane helix near its carboxy terminus (Fig. 4A). Its GFP tagging in transgenic *P. berghei* revealed a clear crystalloid-like localisation in ookinetes (Fig. 4D), indicating that this protein indeed resides in the crystalloids.

Although many proteins in the LAP3 interactome constituted known or novel crystalloid constituents, some proteins have other reported subcellular destinations including osmiophilic bodies [G377 (PBANKA_1463000) [37]]; micronemes [PSOP7 (PBANKA_1353400) [35]]; and the plasma membrane [P47 (PBANKA_1359700) [38]; P25 (PBANKA_0515000) and P28 (PBANKA_0514900) [39]] (Table 1, Table S2). One explanation is that these non-crystalloid proteins interact transiently with the LAP complex whilst in the ER, and have been pulled down from contaminating zygotes that did not complete transformation into ookinete. In support for this, several putative ER-resident ‘housekeeping’ proteins with roles in protein folding were also found enriched in the crosslinked samples, including Hsp70/BiP (PBANKA_0818900), endoplasmin/Hsp90 (PBANKA_1437300), Hsp110 (PBANKA_1357200), HspJ2 (PBANKA_0938300) and two putative protein disulphide isomerases (PBANKA_0942500 and PBANKA_0702800) (Table S1).

## 4 Discussion

Affinity purification combined with mass spectrometry (AP-MS) is a well-established technology for determining complexes and interactomes of a target protein, but is often hampered by non-specific protein contamination that can cause considerable background and complicate interpretation of outcomes [40]. We have used this technology here to determine a putative interactome of LAP3, an established protein of the crystalloid organelle in malaria parasites, and have included several refinement steps to reduce background. Refinements were based on two concepts: (*i*) most protein interactions are weak or transient and will only be identified through *in vivo* crosslinking; (*ii*) most proteins in the organelle share a similar expression and subcellular trafficking strategy. This approach allowed us to determine a putative LAP3 interactome of some 50 proteins (Table 1), which we show is enriched in both known and novel crystalloid constituents. Background proteins identified from our negative LAP3-KO controls were also removed by our bioinformatics filters (Table S1), indicating that the latter were successful in depleting non-specific interacting proteins. Contaminants were further eliminated by quantitative comparison of crosslinked *vs* non-crosslinked samples (Fig. 1A). This step assumes that common contaminants are affinity-purified from both crosslinked and non-crosslinked samples and cancel each other out. By contrast, putative interactors are likely to be enriched in the crosslinked samples and can be identified accordingly, as shown previously for the known interactors LAP4, LAP5 and LAP6 [7]. The repertoire of proteins that are present in the ER at the same time as LAP3 far exceeds the number of proteins in the interactome, indicating that false identification of contaminants that interact ‘accidentally’ with the LAP3 complex was low.

Our findings indicate that many crystalloid proteins physically interact to form an intricate protein complex that extends beyond the six LAPs. What could be the biological significance of this? The crystalloid is a short-lived organelle that forms in ookinetes by coordinated assembly of small ER-derived vesicles, a process that is itself dependent on the synthesis of some of its protein constituents like the LAPs, NTH, and the S-acyl transferase DHHC10 [2,4,16,17]. Crystalloid proteins are trafficked *via* the ER, but specific sorting signals for the organelle have not been identified [10]. One explanation is that proteins destined for the organelle already interact in the ER to ensure that they are trafficked together, thus eliminating the need for individual proteins to possess specific crystalloid targeting signals. Interactions between LAPs already occur before crystalloid formation [7,8]. The formation of a ‘crystalloid protein complex’ in the ER could also explain the phenocopy of null mutants of structurally and functionally unrelated crystalloid proteins, including DHHC10, NTH and the LAPs [[1], [2], [3], [4],^16^,^17^,^41^]: protein interactions within this complex could be highly constrained, and the removal or structural alteration of certain protein components could therefore compromise complex formation or functionality, in turn affecting crystalloid biogenesis and the downstream process of sporogony. Structural modifications of LAP family members have indeed been shown to change their ability to interact with one another [7] and to impact on crystalloid biogenesis [2,4,7,15]. Our recent demonstration that NTH structural knockout parasites do not form crystalloids, while structurally intact but enzymatically inactive NTH is able to support crystalloid biogenesis [17], also supports this hypothesis.

Notable among the new crystalloid proteins identified in this study are two groups of proteins that, like the LAPs, share protein domains: CPW-WPC domain- and PHL domain-containing proteins. The distribution of both these domains among organisms matches that of the LCCL domain and is largely restricted to apicomplexans and chromerids. PH domains are common in eukaryotic proteins and are implicated in binding to phosphatidylinositol lipids within biological membranes, and to several proteins such as protein kinase C and heterotrimeric G proteins [42]. However, we argue that the distant homology of the PHL domain reported here (Fig. 1) with the archetypal PH domain, combined with its unique distribution among organisms, more likely points to unique functions of this protein family specific to apicomplexan parasites. Our GFP tagging of PHL protein PBANKA_0704900 confirmed a localisation in the crystalloid (Fig. 2) in agreement with that of its *P. yoelii* orthologue [32]. However, the crystalloid localisation of PHL protein PBANKA_0417200 shown here (Fig. 2) disagrees with its previously reported localisation on the plasma membrane of *P. berghei* ookinetes [43]. One explanation for this discrepancy is that the earlier study employed immunofluorescence using antibodies raised against the recombinantly expressed antigen to localise the protein. This could have resulted in non-specific binding to the ookinete surface, which is known for its strong adhesive properties [39]. Kou and colleagues reported a loss-of-function phenotype for PBANKA_0417200 consisting of only modest reductions (1.5- to 2-fold) in gametocyte formation, ookinete conversion and oocyst numbers [43]. Moreover, no loss-of-function phenotype was found associated with knockout of the PBANKA_0704900 orthologue in *P. yoelii* [32]. Collectively, these findings indicate that the PHL domain-containing proteins have redundant functions, at least at individual level. The function of the CPW-WPC domain is also unknown. The entire *cpw-wpc* gene family in *Plasmodium* is translationally silenced in gametocytes and activated during ookinete formation, suggesting specific roles in parasite transmission [33]. The crystalloid localisation of CPW-WPC protein PBANKA_1352500 as determined by GFP tagging in live parasites [33] disagrees with the reported surface localisation of its *P. yoelii* orthologue in zygotes and ookinetes as was determined by indirect immunofluorescence with antibodies raised against recombinant antigen [34]. This discrepancy could reflect differences between these parasite species, or between the different methods used to determine the proteins' localisation. The *P. yoelii* orthologue of PBANKA_1352500 did not display a loss-of-function phenotype [34], indicating that there is also functional redundancy within the CPW-WPC domain-containing protein family.

The recent genome-scale barcode study used to analyse the phenotypes of over 1300 *P. berghei* genes through the life cycle is, as a result of heterozygous rescue, poorly suited to identify genes that are essential during the diploid and polyploid life cycle stages (*i.e.* zygotes, ookinetes and oocysts) [36]. Nonetheless, cross-referencing the proteins in our LAP3 interactome with the loss-of-function phenotype for midgut oocyst to salivary gland sporozoite transition (Table 1), indicates that genes with essential roles in sporogony can potentially be identified despite the real loss-of-function phenotypes observed with the individual knockout lines being masked. This is best illustrated by NTH and PSOP13 null mutants, which still show significant reductions in salivary gland sporozoite numbers in spite of heterozygous rescue (Table 1). However, when we used this analysis to assess null mutants of the LAPs, which are also known to have highly impaired sporogony, only LAP2 and LAP5 passed this screen (reductions of 1.2-, 4-, 1.9- and 3-fold, respectively, for LAP1, LAP2, LAP4 and LAP5. LAP3 and LAP6 were not assessed) [36] (Table 1). The latter exemplifies the known limitations of the barcode screen for identifying genes that are either important for sporozoite formation, or for their colonization of the salivary glands [36]. In total, our LAP3 interactome contains 11 proteins (including LAP5, NTH and PSOP13) that display greater than 2-fold reductions in midgut oocyst to salivary gland sporozoite transition using the high throughput null mutant screen, of which 8 values have sufficient power to be statistically significant (Table 1). One of these is the TPM domain protein PBANKA_1104100, which we here demonstrate by GFP tagging to be crystalloid-resident (Fig. 4). In fact, the latter protein is a structural paralogue of PBANKA_0720900 that was reported to be crystalloid-resident and required for sporogony in a previous study [41]. In *Arabidopsis,* phosphatase activity was demonstrated *in vitro* for a TPM domain-containing protein of the thylakoid lumen [44], suggesting that these TPM proteins may constitute crystalloid-specific protein phosphatases.

Information obtained from PlasmoDB reveals that less than half the proteins of the LAP3 interactome have functional annotation (Table S2), which somewhat limits the functional insight that we can gain of the molecular mechanisms that underlie the role of the crystalloid in sporogony. Nonetheless, the new molecules identified will form a useful platform for better understanding these processes through their more in-depth functional characterization in follow-on studies.

The following are the supplementary data related to this article.

Fig. S1 Quality control of AP-MS samples. Fig. S1

Table S1 Parasite proteins identified by AP-MS of *P. berghei* ookinetes expressing GFP-tagged LAP3 with and without *in vitro* crosslinking before pulldown, showing filters and the proteins eliminated by them. Table S1

Table S2 Parasite proteins identified by AP-MS of *P. berghei* ookinetes expressing GFP-tagged TPM2 with and without *in vitro* crosslinking before pulldown, showing filters and the proteins eliminated by them. Table S2
