# Supplementary material for: Plasmodium berghei LAPs form an extended protein complex that facilitates crystalloid targeting and biogenesis
Source: J Proteomics. 2020 Sep 15;227:103925. doi: 10.1016/j.jprot.2020.103925 (PMC7487766; doi:10.1016/j.jprot.2020.103925)
Supplement: Fig. S1 — Quality control of AP-MS samples. [file mmc1.pdf]

A

Sample	Protein count
LAP3/GFP rep 1	135
LAP3/GFP rep 1X	140
LAP3/GFP rep 2	106
LAP3/GFP rep 2X	158
LAP3/GFP rep 3	84
LAP3/GFP rep 3X	119
LAP3/GFP rep 4	25
LAP3/GFP rep 4X	20
LAP3-KO rep 1	119
LAP3-KO rep 2	106
TPM2/GFP rep 1	140
TPM2/GFP rep 1X	189

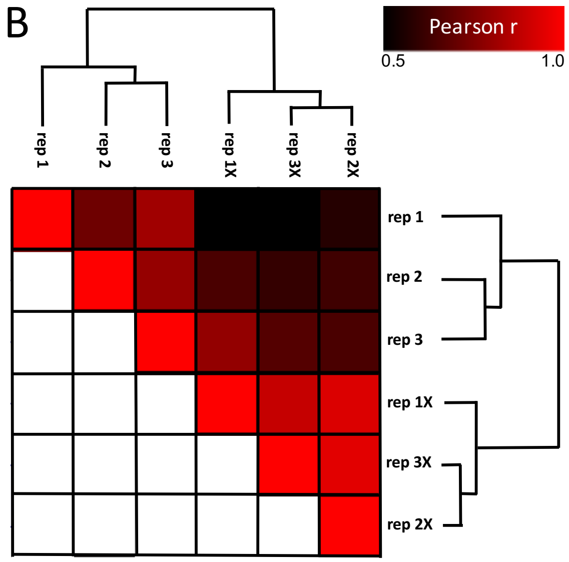

Fig. S1 Quality control of AP-MS samples. **A:** Overview of number of identified *P. berghei* proteins per sample. Samples LAP3/GFP rep 4 (non-crosslinked) and LAP3/GFP rep 4X (crosslinked) were removed from further analysis due to poor proteome coverage compared to other samples. **B:** Hierarchically clustered heat map representation of Pearson cross correlation table showing correlation coefficients of pairwise protein abundance (LOG2 LFQ) comparisons between replicates with Pearson r values ranging between 0.5 and 1. Replicates constitute pulldown samples from LAP3/GFP ookinetes with crosslinking (rep 1X-3X) or without crosslinking (rep 1-3). This analysis demonstrates good correlation between replicates within the sample group without crosslinking (rep 1-3) and within the sample group with crosslinking (rep 1X-3X). Furthermore, the analysis shows that correlation between sample groups is much lower than within sample groups, demonstrating good reproducibility in protein abundance levels within the data set.
